# Age-Related Characteristics of Multipotent Human Nasal Inferior Turbinate-Derived Mesenchymal Stem Cells

**DOI:** 10.1371/journal.pone.0074330

**Published:** 2013-09-16

**Authors:** Se Hwan Hwang, Sun Hwa Park, Jin Choi, Dong Chang Lee, Jeong Hoon Oh, Un Cheol Yeo, Sung Won Kim, Dong Il Sun

**Affiliations:** Department of Otolaryngology-Head and Neck Surgery, The Catholic University of Korea, College of Medicine, Seoul, Korea; University of Milan, Italy

## Abstract

**Background and Objectives:**

Multipotent mesenchymal stem cells (MSCs) represent a promising cell-based therapy for a number of degenerative conditions. Understanding the effect of aging on MSCs is crucial for both autologous therapy development and allogenic donors in older subjects whom degenerative diseases typically afflict. In this study, we investigated the influence of donor age on the characteristics, proliferation, and differentiation potential of *in vitro* cultures of multipotent human turbinated mesenchymal stem cells (hTMSCs) from patients of various age groups.

**Subjects and Methods:**

Twelve patients comprised the four age groups: (I) <20 years, (II) 20–39 years, (III) 40–59 years, and (IV) >60 years. Inferior turbinate tissues were discarded from patients undergoing partial turbinectomy. After isolating hTMSCs, the expression of the hTMSC surface markers CD14, CD19, CD34, CD73, CD90, CD105, and HLA-DR was assessed by FACS analysis, and cell proliferation was assessed using a cell counting kit (CCK)-8. The differentiation potential of hTMSCs was evaluated in osteogenic media by histology and determination of osteoblastic gene expression.

**Results:**

FACS analysis revealed that hTMSCs were negative for CD14, CD19, CD34, and HLA-DR, and positive for CD73, CD90, and CD105, representing a characteristic MSC phenotype, and showed no significant differences among the age groups. Cellular proliferation and osteogenic differentiation potential of hTMSCs also showed no significant differences among the age groups.

**Conclusions:**

We conclude that donor age does not affect the characteristics, proliferation, and osteogenic differentiation potential of hTMSCs. Donor age may be excluded as a criterion in the guidelines for clinical use of the autologous or allogenic transplantation of hTMSCs.

## Introduction

Multipotent mesenchymal stem cells (MSCs) are a population of adult mesenchymal progenitor cells that have the capacity to differentiate into cells of various connective tissue lineages [1]. MSCs are excellent candidates for use in cell therapy because they are accessible, their isolation is straightforward, they can be biopreserved with minimal loss of potency [1], and they have shown no adverse reactions in allogeneic *versus* autologous MSC transplants [2].

Elderly people often suffer from critical-size bone defects due to degenerative or age-related disease [3]. Such bone defects require bone regeneration; and autologous tissues used in implants can be formed in the correct anatomical shape without risk of immunological rejection [3]. Nevertheless, several crucial issues need to be addressed, such as long procedure times and a high proportion of graft-site morbidity among patients. In contrast, MSCs are relatively easy to obtain from bone marrow, adipose tissue, and soft tissue, resulting in minimal morbidity. Therefore, MSCs are of major clinical interest for the development of cell-based strategies to treat musculoskeletal diseases, including bone defects caused by trauma, degenerative disorders, or infection [4]. While many studies have focused on characterizing MSCs, the majority were performed using cells derived from young donors. However, one of the most important arguments for use of MSCs is the possibility of performing autologous or allogeneic transplantations in an aging society. Considering the conditions that are potential indications for cell therapy, it is clear that autologous or allogeneic transfer would often require the isolation and extracorporeal proliferation of cells derived from aged patients. Aging is a highly complex process that affects both cells and extracellular matrix (ECM), leading to general impairment of tissue and organ function [5]. However, there is currently no consensus and widely disparate results have been reported regarding the number of MSCs and their function in aging individuals; some groups found an age-related decline whereas others reported no change [6].

**Figure 1:**
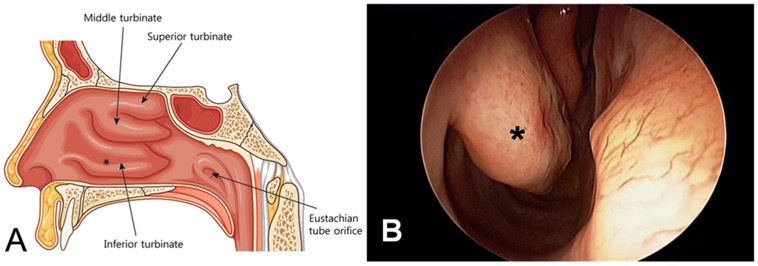
Otolaryngologic finding of the nasal cavity. A: Schematic of the lateral nasal wall of the right nasal cavity. B: Endoscopic visualization of the right nasal cavity. *Inferior turbinate.

In a previous study, we showed that inferior turbinate tissues removed during turbinate surgery, which is most commonly performed with ease under local anesthesia in the field of otolaryngology, could be used as a source of MSCs (Figure 1) [7]. In addition, we reported that MSCs from human inferior turbinate (hTMSCs) showed a relatively high yield and that the passage number did not affect their characteristics. These properties allow expansion of the indications for use of multipotent MSCs. However, age-related functional changes in hTMSCs have not been investigated to date. Considering the effect of aging on MSCs from other sources, we assume that age-related changes in hTMSCs either negatively affect proliferative or osteo-differentiation potential or have no effect. Therefore, understanding the effects of aging on the differentiation potential of hTMSCs will be critical to the success of their therapeutic application in regenerative medicine. In this study, we investigated the influence of donor age on the characteristics, proliferation, and differentiation potential of *in vitro* cultures of multipotent hTMSCs from patients of various age groups.

## Materials and Methods

### Ethics Statement

All studies using hTMSCs were performed after written approval (KC08TISS0341) from the institutional review board of Seoul St. Mary’s Hospital, the Catholic University of Korea and after obtaining written informed consent from the donors themselves. In case of the donors aged 10–19 years, informed consent was obtained from their parents on the behalf of participants. Investigations were conducted according to the principles expressed in the Declaration of Helsinki.

### Donors

Inferior turbinate tissues were discarded from 12 patients undergoing partial turbinectomy. Four groups were studied: (I) three patients aged 10–19 years; (II) three patients aged 20–39 years; (III) three patients aged 40–59 years; and three patients aged 60–75 years.

### Cell Isolation

An identical quantity (0.0366 g) of inferior turbinate tissues was obtained from discarded tissue and washed three to five times with saline solution containing gentamicin (Kukje Pharmaceutical Industries, Sungnam, Korea). To isolate hTMSCs, the inferior turbinate tissue was washed at room temperature three times with antibiotic-antimycotic solution (Gibco, Gaithersburg, MD) and twice with phosphate buffered saline (PBS), and then cut into 1-mm^3^ pieces. The pieces were placed into a culture dish, and the dish was covered with a sterilized glass cover slide. DMEM (Dulbecco’s Modified Eagle Media, Gibco) containing 10% fetal bovine serum (FBS) was added, and the tissues were incubated at 37°C in a 5% CO_2_ atmosphere; the culture medium was changed every two or three days. After 3 weeks of culture, the glass cover slide was removed, and tissues floating in the culture medium were removed by washing. The hTMSCs that had attached to the bottom of the culture dish were detached using 3 ml of 0.25% trypsin in 1 mM EDTA; the cell count was done with ADAM automated cell counter (Digital Bio, Seoul, Korea) [8]. We used the isolated cells cultured to three passages. The cells in three passages were examined for age-related changes in their immunophenotypical characteristics, proliferation, and osteo-differentiation potential.

### hTMSCs Surface Marker Expression

Flow cytometry for assay cell surface markers was conducted. hTMSCs from turbinate were plated into a test tube (BD, Franklin Lakes, NJ) at a density of 1×10^5/^mL and washed three times with wash buffer (PBS and 3% FBS). The cells were incubated for 40 min with saturating concentrations of primary monoclonal antibodies CD14, CD19, CD34, CD73, CD90, CD105, and HLA-DR. After washing three times in buffer and centrifugation at 1200 rpm for 5 min, cells were resuspended in ice-cold PBS and incubated with the secondary antibody for 30 min in the dark at 40°C. Fluorescence was evaluated by flow cytometry using a FACSCalibur instrument (BD), and the data were analyzed using the Cell Quest software (BD). In addition, the expression of the neural crest marker BMP-4, BMP-7, CD49d, p75, SOX9, SOX10, and Slug was assessed by FACS analysis [9], [10].

### Proliferation Assay

To generate growth curves, hTMSCs from the four groups were plated simultaneously into 96-well tissue culture plates at a density of 1500 cells per well. The medium was replaced every 2 days. Cell proliferation was assessed using a cell counting kit (CCK)-8 (Dojindo Laboratories, Kumamoto, Japan), following the manufacturer’s instructions. The culture medium was removed and 100 µL of fresh medium containing 10-µL CCK-8 was added to each well. The cells were then incubated at 37°C for 4 h. Cell viability was monitored for 14 days. The optical density values were determined in at least triplicate against a reagent blank at a test wavelength of 490 nm and a reference wavelength of 630 nm.

### Osteogenic Differentiation Potential of hTMSCs

To induce osteogenic differentiation, hTMSCs were seeded in 12-well tissue culture plates (2×10^4^ cells/well) and incubated in low-glucose DMEM supplemented with 10% FBS, 100 U/ml penicillin, 100 µg/ml streptomycin, 0.1 µM dexamethasone, 50 µM ascorbate-2-phosphate, and 10 mM β-glycerophosphate (all from Sigma). The induction culture was maintained for 4 weeks, and the medium was replaced every 3 days. The cells were then subjected to histological alkaline phosphatase (ALP) and alizarin red S staining, and RT-PCR.

### Histological Studies

Before and after the initiation of osteogenic induction, cells were washed three times with sterile triple-distilled water. Some of the cells were fixed by incubation in 4% paraformaldehyde for 15 min, stained with 2% alizarin red solution (Sigma) for 30 min in the dark, and then washed three times with sterile triple-distilled water. A proportion of the cells was fixed in citrate-acetone-formaldehyde fixation solution (Sigma), washed three times with sterile triple-distilled water, stained with an alkaline dye mixture (nitrite, FRV-alkaline, naphthol AS-Bl alkaline solution; Sigma) for 15 min at room temperature in the dark, and washed three times with sterile triple-distilled water. The cells were counterstained with hematoxylin (Sigma), washed three times with sterile triple-distilled water, and observed under an inverted microscope.

### RNA Extraction and RT-PCR of hTMSCs

Total RNA was extracted from cells cultured for chondrogenic differentiation and osteogenic differentiation using TRIzol® reagent (Invitrogen). RNA was extracted according to the method recommended by the manufacturer (Kontes, Vineland, NJ). For cDNA synthesis, total RNA (2 µg) from each sponge was diluted to a volume of 10 µl in DEPC/DDW and used in a reaction (10 µl) containing 4 µl of 5× first-strand buffer (Invitrogen), 4 µl of DTT (0.1 M, Invitrogen), 1 µl of dNTPs (10 mM, Sigma), 0.5 µg of oligo (dT) 15 primer (Invitrogen), 1 µl of RNase inhibitor (Invitrogen), and 200 units of SuperScript™ II (Invitrogen). The reaction was incubated at 45°C for 60 min. The cDNA was stored at −20°C until needed.

PCR reactions (50 µl) contained GoTaq® Flexi DNA polymerase (Promega), 4 µl of MgCl_2_ (25 mM, Invitrogen), 2 µl of dNTPs (10 mM, Sigma), cDNA template, and primers specific for one of the following: bone morphogenetic protein (BMP)-2, runt-related transcription factor (RunX) 2, bone sialoprotein (BSP), collagen type I (Col1), osteocalcin (OC), osterix (Osx), and osteoponin (OP) (Table 1). The thermal conditions for PCR amplification were: initial denaturation at 94°C for 3 min, followed by 35 cycles of 94°C for 30 s, 53°C for 30 s, and 72°C for 30 s, with a final extension at 72°C for 5 min. PCR products were separated in 2% agarose (Sigma) gels in 1× Tris-acetate-EDTA buffer containing ethidium bromide.

**Table 1:** Oligonucleotide primer sequences used for reverse transcriptase polymerase chain reaction (RT-PCR).

Gene	Primer	Sequence
BMP2	forward primer (FP)	5′-TTG CGG CTG CTC AGC ATG TT -3′
	reverse primer (RP)	5′- CAT CTT GCA TCT GTT CTC GGA A-3′
Osx	forward primer (FP)	5′-CTT CAG TCT TCC CAA CTT CTT ACA C-3′
	reverse primer (RP)	5′ -ACA AAT TGG GTT AGC TAC ATC TCT G-3′
BSP	forward primer (FP)	5′ -GCT CAG CAT TTT GGG AAT GGC -3′
	reverse primer (RP)	5′ -CTG CAT TGG CTC CAG TGA CAC -3′
Runx2	forward primer (FP)	5′- GTG GAC GAG GCA AGA GTT TCA -3′
	reverse primer (RP)	5′- TGG CAG GTA GGT GTG GTA GTG -3′
COL1	forward primer (FP)	5′ - TGA CGA GAC CAA GAA CTG -3′
	reverse primer (RP)	5′ - CCA TCC AAA CCA CTG AAA CC -3′
OC	forward primer (FP)	5′ - ATG AGA GCC CTC ACA CTC CTC -3′
	reverse primer (RP)	5′- CGG GCC GTA GAA GCG CCG ATA -3′
OP	forward primer (FP)	5′- CAG TGA TTT GCT TTT GCC TGT TTG -3′
	reverse primer (RP)	5′-GGT CTC ATC AGA CTC ATC CGA ATG -3′

### Statistical Analyses

Statistical analyses were conducted using the SPSS 18 statistical software (SPSS Inc., Chicago, IL, USA). The statistical significance of differences among groups was determined by one-way analysis of variance (ANOVA). A *p* value <0.05 was considered to indicate significance.

## Results

### Surface Marker Expression

Surface markers on the expanded cells were evaluated by flow cytometry. hTMSCs from all age groups were negative for hematopoietic markers (CD14, CD19, CD34, and HLA-DR) and positive for MSC markers (CD73, CD90, and CD105). The expression levels of specific surface proteins were similar among the four groups (Table 2). The expressions of the neural crest markers such as BMP-4, BMP-7, and CD49d in four groups were mainly lower than ten percents and similar among the four groups. In addition, p75, SOX9, SOX10, and Slug were lower than one percent in the all groups. These results showed that the hTMSCs would be composed of mainly MSCs (Table 3).

**Table 2:** Effects of donor age on the immunophenotypic characteristics of hTMSCs.

Surface marker Expression	CD14	CD19	CD34	CD73	CD90	CD105	HLA-DR
Group I	0.03%	0.00%	0.11%	99.24%	99.81%	99.66%	0.1%
Group II	0.02%	0.00%	0.02%	98.22%	99.79%	99.40%	0.6%
Group III	0.05%	0.04%	0.04%	99.23%	99.76%	99.16%	0.04%
Group IV	0.01%	0.03%	0.00%	98.22%	99.72%	99.57%	0.2%

**Table 3:** Effects of donor age on the neural crest markers of hTMSCs.

Surface Marker Expression	BMP-4	BMP-7	CD49d	p75	SOX9	SOX10	Slug
Group I	1.92%	2.35%	1.46%	0.07%	0.72%	0.83%	0.83%
Group II	3.50%	3.38%	11.19%	0.09%	0.54%	0.10%	0.57%
Group III	2.35%	2.08%	3.10%	0.05%	0.37%	0.11%	0.38%
Group IV	3.83%	2.38%	8.92%	0.04%	0.26%	0.01%	0.27%

### Proliferation Assay

**Figure 2:**
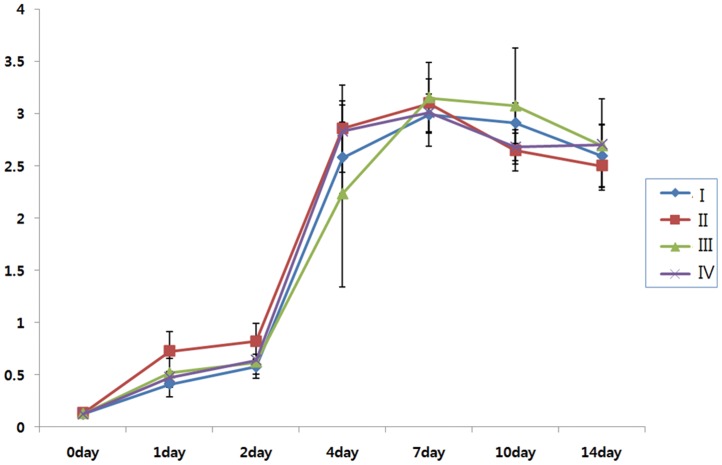
Proliferation of hTMSCs according to age group. Cellular proliferation was monitored over a period of 14 days. hTMSCs from all groups exhibited rapid proliferation from day 2 to 4; hTMSCs from group (II) expanded more rapidly than those from the other groups. However, there were no statistically significant differences in proliferation rate among the groups.

Cell proliferation was monitored over a period of 14 days. hTMSCs from all age groups were in stationary phase during the first 2 days. From day 2 to 4, the cells underwent logarithmic growth, during which hTMSCs from group II expanded more rapidly than those from the other groups (Figure 2). Then, cells from all age groups entered a lag phase and showed a pattern of decreasing cell numbers during the following days. However, there were no statistically significant differences in proliferation rate among the groups.

### Osteogenic Differentiation Potential

**Figure 3:**
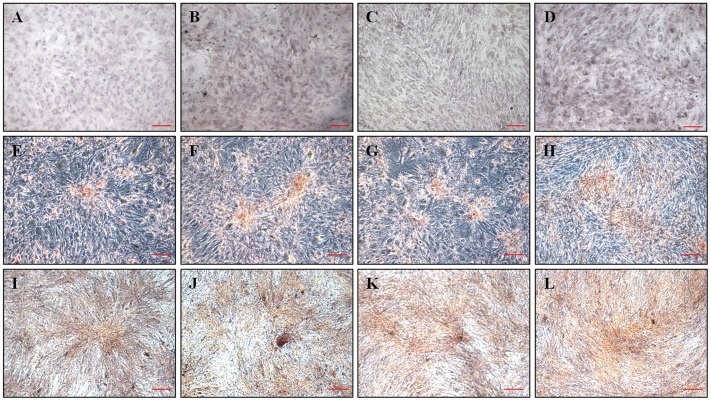
Effect of donor age on the osteogenic differentiation potential of hTMSCs. Cells were cultured in osteogenic induction medium. Cells aggregated, formed nodules, and accumulated calcium deposits over a 2-week period. A–D: Alkaline phosphatase staining of hTMSCs cultured from each age group before osteogenic induction. E–H: Alkaline phosphatase staining of hTMSCs cultured from each age group after osteogenic induction. Alkaline phosphatase activity, which is indicative of osteoblastic differentiation, is shown as red staining. I–L: Alizarin red staining of hTMSCs cultured from each age group after osteogenic induction. (A, E, and I: group I; B, F, and J: group II; C, G, and K: group III; D, H, and L: group IV). Alizarin Red staining was used to detect precipitated calcium salt, which is a marker of differentiation. Scale bars: 100 µm. Visual assessment data demonstrated that hTMSCs cultured from donors of all age groups showed the alkaline phosphatase activity increase after osteogenic induction and similar levels of alkaline phosphatase and Alizarin Red staining.

Before osteogenic induction of hTMSCs, cells in four groups showed the negative staining for ALP. After osteogenic induction, hTMSCs isolated from the four age groups showed direct evidence of intracellular osteogenic differentiation and extracellular mineral deposition, as detected by ALP and alizarin red S staining (Figure 3). After 14 days of culture, osteogenically induced hTMSCs reached near confluence in all four groups, and stained intensely for ALP and alizarin red S. No differences in visible color density were observed among the four groups.

**Figure 4:**
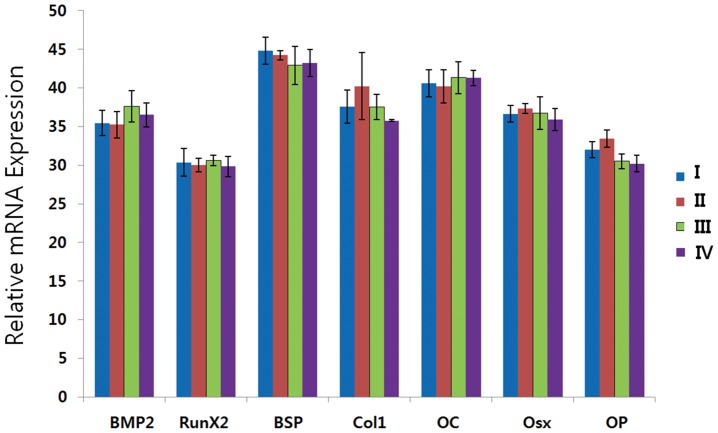
RT-PCR analysis of osteoblast-specific genes expression during 2 weeks of culture in osteogenically differentiated hTMSCs. Bone morphogenetic protein-2 (BMP-2), runt-related transcription factor 2 (Runx2), bone sialoprotein (BSP), type I collagen (Col1), osteocalcin (OC), osterix (Osx), and osteopontin (OP) mRNA expression were analyzed. The experiment was performed in triplicate for each sample. Differences among the groups were evaluated by one-way analysis of variance (ANOVA; *p*<0.05). Except for OP, there were no significant differences in expression levels among the groups.

RT-PCR was used to determine the mRNA levels of osteoblast-specific genes encoding BMP-2, Runx2, BSP, Col1, OC, Osx, and OP in osteogenically induced hTMSCs at the end of the culture period. The expression levels of osteoblast-specific genes (BMP-2, Runx2, BSP, Col1, OC, and Osx) did not differ among the four groups (except for OP) (Figure 4).

## Discussion

Tissue engineering has the potential to create an abundant quantity of tissue from a small donor specimen [11]. Reconstruction of the bony skeleton using tissue-engineered constructs requires autologous cells or MSCs in a scaffold that directs their organization. However, use of autogenous osteoblasts or osteocytes is limited by their availability and activity. The ability to engineer tissues from autologous or allogenous MSCs may overcome that limitation in regenerative medicine [12]. In particular, human MSCs have been obtained from bone marrow, adipose tissue, and soft tissue. Thus, MSCs are of considerable clinical interest in the development of cell-based strategies for the treatment of musculoskeletal diseases [13].

Although bone engineering with biomaterials seeded with MSCs is effective in replacing lost bone, it was reported recently that bone reconstruction with bone marrow MSCs is effective for long bones but not effective for jaw reconstruction. This is due to the fact that the facial skeleton is derived from the cranial neural crest and the appendicular skeleton is derived from the mesoderm. Although MSCs with a facial origin can differentiate into osteoblasts after implantation into the facial skeleton, MSCs from bone marrow fail to differentiate into osteoblasts when implanted into facial bone [8]. These data emphasize the importance of the origin of MSCs used for tissue repair [14]. Neural crest-derived stem cells (NCSCs) in the head region can be found in a number of adult organs and tissues, such as dental pulp, periodontal ligament, skin, and hair follicles. However, these stem cells are not ideal candidates for clinical use due to external scarring, painful procedures, and limited source material [15]. The human respiratory mucosa of the inferior turbinate was recently reported to contain a novel NCSC population. In contrast to other sources, MSCs from the inferior turbinate could be harvested by a simple and minimally invasive procedure from a donor site that heals rapidly without morbidity or scar formation, which would reduce the pain and morbidity suffered by MSC donors [7]. In addition, MSCs from the inferior turbinate could be isolated in an age-independent manner from individuals aged 4–76 years [15]. Therefore, hTMSCs may be an ideal candidate for facial bone reconstruction.

Several factors have been shown to influence the osteoblastic differentiation of MSCs, such as osteogenic factors and donor age. In a previous study, it was demonstrated that hTMSCs are able to differentiate into an osteogenic phenotype under common osteogenic stimulation. In addition, hTMSCs are not only highly proliferative but they also have osteogenic differentiation potential independent of additional osteogenic ingredients, such as Vit D3 and BMP-2, as compared with bone marrow or adipose tissue-derived MSCs (BMSCs and AdMSCs) [13]. Therefore, osteo-differentiation of hTMSCs could be induced by use of inorganic bone-formation stimulating factors without the need for gene transfection using viral vectors. However, for development of an effective bone regeneration method using hTMSCs, the effects of donor age on the hTMSCs should be evaluated, as has been done for BMSCs and AdMSCs. The aim of the present study was to determine whether the characteristics, proliferation, and osteo-differentiation potential of hTMSC *in vitro* cultures are influenced by donor age by comparing the cell surface marker expression, growth curves, and histological characteristics of hTMSCs derived from various age groups.

hTMSCs can be characterized by FACS analysis of specific surface proteins, such as CD73, CD90, and CD105. After isolation, hTMSCs are negative for hematopoietic factors such as CD14, CD19, CD34, and HLA-DR. Using FACS analysis, we found no significant age-related changes in cell surface expression. However, Stolzing *et al*. reported an age-related decline in the expression of CD90 and CD105, and an increased expression of CD44 in BMSCs [16]. MSC markers such as specific surface proteins are commonly used to isolate, enrich and characterize MSCs *in vivo* and *in vitro*. This information is of vital importance for the analysis of MSC numbers in relation to donor age when MSCs are isolated using one or several MSC markers [16]. Aging did not seem to be correlated with the expression of several other markers of hTMSCs (*e*.*g*., CD90 and CD105), which may lead to consistencies when comparing reports of changes in hTMSCs numbers from independent research groups. Considering the effects of passage number in both previous reports and our findings, unlike BMSCs, these markers are reliable for characterizing hTMSCs in studies of the effects of age and passage number [7].

Growth curves of most stem cell types, including BMSCs and AdMSCs, appear to reach a plateau on the sixth or seventh day [17]. However, the growth curves we report here plateaued more rapidly. The plateau phase occurred within 4 days and was followed by a long lag stage, which may indicate contact inhibition or decreased proliferation due to reaching confluence. This result is similar to that of our previous study [7], which indicates that hTMSCs have a greater growth ability than BMSCs and AdMSCs. In addition, hTMSCs showed a high proliferative ability irrespective of donor age. This differs from BMSCs, those isolated from the youngest donor group grew faster than those isolated from the older groups [3].

Three distinct phases of MSC differentiation and bone formation are known: proliferation, extracellular matrix maturation and matrix mineralization [18]. The first stage consists of days 1 to 4, when a peak in the number of cells is observed. This is followed by early cell differentiation from days 5 to 14, which is characterized by the transcription and protein expression of Col1 and alkaline phosphatase (ALP), implying that Col1 and ALP are early markers of the osteoblast lineage. The final stage, from days 14 to 28, results in high expression of OP, BSP, and OC, followed by calcium and phosphate deposition [19]. OP and BSP are early indicators of osteogenic differentiation used to identify osteoprogenitor cells during early osteogenesis, prior to mineralization or osteocalcin expression. OC is thought to indicate a more mature osteoblastic phenotype of the osteocyte [20]. Runx2 and Osx are primary osteoblast-specific transcription factors for osteoblastic differentiation, [21] and positively regulate OC and BSP expression [22]. BMP-2 is known to regulate the mechanism upstream of Runx2 in osteogenic differentiation [23]–[25].

The osteogenic capacity of hTMSCs was assessed after osteogenic differentiation. Comparing the staining for ALP before and after osteogenic induction, hTMSCs after induction demonstrated the ALP increase. After culturing in osteogenic media for 14 days, the induced hTMSCs formed an extensive network that stained densely positive for ALP and a calcified extracellular matrix that stained positively for alizarin red. There were no significant differences in visual assessment of ALP and alizarin red staining among hTMSCs derived from the four age groups. To further evaluate the osteogenic capacity of hTMSCs, the expression levels of osteoblast-related genes were determined by RT-PCR. Although there were significant differences in the expression of OP among the four groups, the expression levels of the other osteoblast-associated genes showed no differences among the four groups. These findings suggest that aging had no significant effect on the osteogenic capacity of hTMSCs.

Hauser *et al.* [15] reported the isolation of neural crest related stem cells from the same turbinate tissue which was verified with the expression of the markers for the neural crest as p75, SOX9, SOX10, and Slug. Those cells formed neurospheres under serum-free conditions in the presence of FGF-2 and EGF [15], which was different from neurospheres induction that incubated in neurobasal medium with neurogenic supplements in our previous study [26]. In this study, the expressions of other neural crest markers (BMP-4, BMP-7, and CD49d) as well as p75, SOX9, SOX10, and Slug were assessed by FACS analysis and mainly lower than ten percents (BMP-4, BMP-7, and CD49d) or one percent (p75, SOX9, SOX10, and Slug). It could mean that the mesenchymal stromal cells from the nasal inferior turbinate would be composed of MSC and neural crest related stem cells. In addition, most of the studies describing the bone marrow derived mesenchymal stromal cells on low passages (<4) mainly contained MSC and less than 10% of neural crest related stem cells [27], which would support that the hTMSCs in our study would be composed of mainly MSCs.

This study is the first to demonstrate that hTMSCs express MSC-specific surface proteins, are highly proliferative, and differentiate into cells with the osteogenic phenotype irrespective of donor age. These results facilitate an understanding of the characteristics of hTMSCs, and may have a considerable impact on clinical medicine. However, because the molecular and/or biochemical basis of the proliferation and differentiation of hTMSCs remains unknown, further studies at the cellular, biochemical and molecular levels should be performed to permit effective control of hTMSC proliferation and differentiation.

## Conclusion

These data suggest that donor age does not affect the characteristics, proliferation, or osteogenic differentiation potential of hTMSCs, allowing expansion of the use of multipotent mesenchymal stromal cell donors. In particular, in comparison with BMSCs, which exhibit declines in proliferation and osteogenic potential with donor age, this will facilitate development of an effective method for tissue regeneration due to the availability of an unlimited quantity of tissue which was previously discarded. Moreover, these findings can be used to establish a custom tissue and cell bank for patients with a variety of diseases.
